# A Systematic Review of the Frequency of Neurocyticercosis with a Focus on People with Epilepsy

**DOI:** 10.1371/journal.pntd.0000870

**Published:** 2010-11-02

**Authors:** Patrick C. Ndimubanzi, Hélène Carabin, Christine M. Budke, Hai Nguyen, Ying-Jun Qian, Elizabeth Rainwater, Mary Dickey, Stephanie Reynolds, Julie A. Stoner

**Affiliations:** 1 Department of Biostatistics and Epidemiology, University of Oklahoma Health Sciences Center, Oklahoma City, Oklahoma, United States of America; 2 Department of Veterinary Integrative Biosciences, College of Veterinary Medicine, Texas A&M University, College Station, Texas, United States of America; 3 National Institute of Parasitic Diseases, Shangai, People's Republic of China; Université de Limoges, France

## Abstract

**Background:**

The objective of this study is to conduct a systematic review of studies reporting the frequency of neurocysticercosis (NCC) worldwide.

**Methods/Principal Findings:**

PubMed, Commonwealth Agricultural Bureau (CAB) abstracts and 23 international databases were systematically searched for articles published from January 1, 1990 to June 1, 2008. Articles were evaluated for inclusion by at least two researchers focusing on study design and methods. Data were extracted independently using standardized forms. A random-effects binomial model was used to estimate the proportion of NCC among people with epilepsy (PWE). Overall, 565 articles were retrieved and 290 (51%) selected for further analysis. After a second analytic phase, only 4.5% of articles, all of which used neuroimaging for the diagnosis of NCC, were reviewed. Only two studies, both from the US, estimated an incidence rate of NCC using hospital discharge data. The prevalence of NCC in a random sample of village residents was reported from one study where 9.1% of the population harboured brain lesions of NCC. The proportion of NCC among different study populations varied widely. However, the proportion of NCC in PWE was a lot more consistent. The pooled estimate for this population was 29.0% (95%CI: 22.9%–35.5%). These results were not sensitive to the inclusion or exclusion of any particular study.

**Conclusion/Significance:**

Only one study has estimated the prevalence of NCC in a random sample of all residents. Hence, the prevalence of NCC worldwide remains unknown. However, the pooled estimate for the proportion of NCC among PWE was very robust and could be used, in conjunction with estimates of the prevalence and incidence of epilepsy, to estimate this component of the burden of NCC in endemic areas. The previously recommended guidelines for the diagnostic process and for declaring NCC an international reportable disease would improve the knowledge on the global frequency of NCC.

## Author Summary

Neurocysticercosis (NCC) is a parasitic infection of the brain caused by the tapeworm *Taenia solium*, which infects humans and pigs. There have been increasing case reports and epidemiological studies on this disease, but its global frequency has never been determined, partly due to the fact that blood tests are not very good for the diagnosis of NCC. We present here a systematic review of the literature on the frequency of NCC diagnosed with neuroimaging worldwide. Overall, 565 articles were retrieved and 290 (51%) selected for further review. Of those, only 26 had information valid enough to estimate the frequency of NCC in various populations. Only one study estimated the prevalence of NCC in the general population. The most striking finding was that the proportion of NCC among persons with epilepsy was very consistent and estimated at 29.6% (95%CI: 23.5%–36.1%) from 12 studies conducted in Latin America, Sub-Saharan Africa and Southeast Asia. A reinforcement of the suggested universal guidelines for the diagnostic process, declaring NCC an international reportable disease and standardizing procedures for data collection could improve our understanding of the frequency of NCC worldwide and hence its global burden.

## Introduction

Scientific evidence is instrumental to improving global public health, as health policies should be based on accurate and meaningful data. In early 1990, the first Global Burden of Diseases (GBD) study, commissioned by the World Bank, was launched to develop a method to estimate and compare the burden of 107 diseases and injuries around the world. A standardized indicator, the “Disability Adjusted Life Years” (DALY) method, was developed for this purpose [1]–[2]. Unfortunately, only a few neglected tropical diseases (NTD) and no neglected tropical zoonoses were taken into account in the original GBD study.

Neglected tropical diseases are a public health issue worldwide and especially in developing countries, where risk factors for their transmission are common [3]. These conditions tend to affect the poorest of the poor, which has led to limited research interest and investments for these infections. The few research initiatives that have been undertaken to estimate the burden of NTDs have been criticized for grossly underestimating their global impact [4]–[6]. In addition, the burden of several zoonotic NTDs, such as *Taenia solium* cysticercosis, has never been estimated.

**Figure 1:**
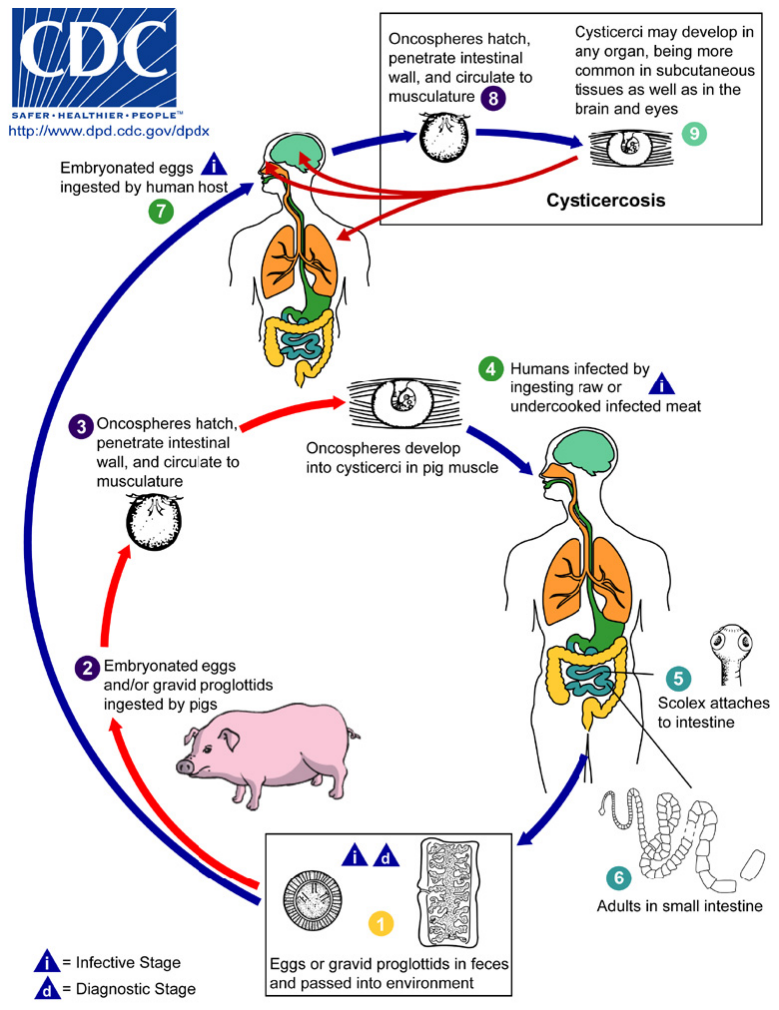
Life cycle of *Taenia solium* cysticercosis (source: CDC-DPDx). Cysticercosis is an infection of both humans and pigs with the larval stages of the parasitic cestode, *Taenia solium*. This infection is caused by ingestion of eggs shed in the feces of a human tapeworm carrier (1). Pigs and humans become infected by ingesting eggs or gravid proglottids (2), (7). Humans are infected either by ingestion of food contaminated with feces, or by autoinfection. In the latter case, a human infected with adult *T. solium* can ingest eggs produced by that tapeworm, either through fecal contamination or, possibly, from proglottids carried into the stomach by reverse peristalsis. Once eggs are ingested, oncospheres hatch in the intestine (3), (8) invade the intestinal wall, and migrate to striated muscles, as well as the brain, liver, and other tissues, where they develop into cysticerci (9). In humans, cysts can cause serious sequellae if they localize in the brain, resulting in neurocysticercosis. The parasite life cycle is completed, resulting in human tapeworm infection, when humans ingest undercooked pork containing cysticerci (4). Cysts evaginate and attach to the small intestine by their scolex (5). Adult tapeworms develop, (up to 2 to 7 m in length and produce less than 1000 proglottids, each with approximately 50,000 eggs) and reside in the small intestine for years (6). (This life cycle is available online at URL: http://www.dpd.cdc.gov/dpdx/HTML/Cysticercosis.htm).

The lifecycle of *T. solium* is illustrated in Figure 1. Humans acquire cysticercosis when ingesting food that has been contaminated with infected feces or through auto-infection. Neurocysticercosis (NCC), which occurs when the larvae of *T. solium* migrate to the brain, has been reported as the most frequent helminthic infection of the central nervous system (CNS) [5], [7]. Yet, there have been very few studies conducted to estimate the prevalence of NCC. This is mostly due to the fact that NCC can only be diagnosed with certainty through neuro-imaging or autopsy. Hence, the frequency of sequelae following infection with the larval stages of *T. solium* remains largely unknown [8]. Exact data on the worldwide frequency of CNS infections with cysticercosis is lacking [9].

The primary objective of this study was to conduct a systematic review of the literature to gather data on estimates of NCC frequency between 1990 and 2008 by age group and region. A secondary aim was to estimate the proportion of people living with epilepsy (PWE) who have NCC. This study was commissioned by the World Health Organization's Foodborne Disease Burden Epidemiology Reference Group (FERG). The FERG is the World Health Organization's advisory body to estimate the global burden of foodborne diseases.

## Methods

A systematic search of the literature was conducted to gather valid information on the frequency (prevalence proportion, incidence rate, or proportion among a specific population) of NCC between 1990 and 2008.

### Searching

The search strategy was conducted in three phases. In phase I, PubMed, Commonwealth Agricultural Bureau (CAB) Abstracts, and 23 international databases (Table 1) were screened for articles published from January 1, 1990 to June 1, 2008. Searches were restricted to languages that at least two of the members of the team could read and understand, namely English, French, Italian, Romanian, German, Chinese, Spanish and Portuguese. In PubMed, our search strategy included the Medical Search Heading (MeSH) terms: “Neurocysticercosis/epidemiology”. Because CAB Abstracts and the international search engines did not allow for searches using MeSH terms, they were queried using the following keywords: “*Taenia solium*”, “taeniasis”, “cysticercosis”, and “neurocysticercosis”. Only one copy of duplicated documents was kept for analysis. Studies were selected that included original epidemiological data on NCC frequency. Books and conference abstracts were excluded because they were unlikely to present original data or to have sufficient details on methods to judge the validity of the study. Dissertations, theses, and memoirs were included. Moreover, due to the under-representation of publications from Sub-Saharan Africa, three unpublished studies (at that time) were reviewed in addition to one paper published online in November 2008 [10]. One unpublished study on the incidence rate of NCC from Oklahoma was also included since very few publications reported incidence rates of NCC (Thompson J, unpublished data).

**Table 1:** Results of search strategies on neurocysticercosis epidemiology published between 1990 and 2008 (June).

Database	Link	Hits
**African Women's Bibliographic Database**	http://www.africabib.org/women.html	0
**African Journal Online**	http://www.ajol.info/	1
**Article published after June 2008**		1
**Articles from other search**		4
**Bibliothèque Santé Tropicale**	http://www.santetropicale.com/resume/catalogue.asp	1
**Bioline International**	http://www.bioline.org.br/journals	0
**CAB abstracts**	http://www.cabdirect.org/	209
**China Knowledge Infrastructure (CHKI)**	http://www.global.cnki.net/	33
**CUIDEN**	http://www.doc6.es/index/	0
**Ecole Nationale de la Santé Publique**	http://www.bdsp.tm.fr	5
**Find Articles**	http://www.findarticles.com/	0
**GreySource**	http://www.greynet.org/greysourceindex.html	0
**Ind Med**	http://indmed.nic.in	19
**Institute of Tropical Medicine (ITM) in Antwerp Belgium**	http://lib.itg.be:8000/webspirs/start.ws	2
**IranMEDEX**	http://www.iranmedex.com/english/index.asp	1
**Japan Science and Technology Information Aggregator**	http://www.jstage.jst.go.jp/browse/	0
**KoreaMed**	http://www.koreamed.org/SearchBasic.php	0
**Médecine et Pharmacie de l'Université, Bamako**	http://www.keneya.net	0
**MetaBase**	http://infolac.ucol.mx/boletin/14_1/innovaciones1.html	4
**OpenMED@NIC**	http://openmed.nic.in	0
**PhD Dissertation**		1
**PUBMED**	http://pubmed.org	240
**Science Research Portal**	http://scienceresearch.com/search/index.php	6
**The Trials Register of Promoting Health Interventions (TRoPHI)**	http://eppi.ioe.ac.uk/EPPIWeb/home.aspx?Control=Search&SearchDB=trials&page=/hp/	0
**Unpublished data or in press**		3
**WHO Africa**	http://indexmedicus.afro.who.int/	0
**WHO Eastern Mediter**	http://www.emro.who.int/HIS/VHSL	9
**WHO South-East Asia**	http://www.hellis.org	2
**WHO Western Pacific**	http://www.wpro.who.int/information_sources/library_services/wprim.htm	34

Another component of this project was to assess the proportion of sequelae associated with NCC (details reported elsewhere). This search led to the finding of four additional studies which described the proportion of NCC among people with epilepsy and seizures [11]–[12] and among children with partial seizures [13]–[14].

### Selection

The inclusion and exclusion criteria were defined *a priori*. In phase I, all documents retrieved were screened based on title and abstract. The exclusion criteria for phase I were: 1) wrong agent (for example, *T. saginata*); 2) animal data only; 3) no original data on the frequency of NCC; 4) case series with less than 20 participants; 5) review article without original data; and 6) editorials or letters to the editors without original data. Documents without abstracts were included in the next phase. All eligible documents after phase I were obtained in full. Each full document was read and reviewed by at least two investigators and subjected to the two subsequent phases of review. Phase II and III corresponded to a qualitative and quantitative appraisal of the information, respectively. The exclusion criteria for phase II included all criteria used in phase I in addition to: 1) no neuro-imaging (CT-scans or MRI) or autopsies used for the diagnosis of NCC; 2) high potential for selection bias (study of volunteers, study population obviously more at risk of NCC than the target population); or 3) all available data from before 1990 or after 2008 (except for sub-Saharan Africa where two studies published in 2009 were included). The data from documents included after phase II were extracted in phase III.

### Data extraction

During phase I, the full reference of each article, the country where the study was conducted, the decision on inclusion for phase II, the reason for exclusion (if applicable) and the language of the document were entered into an Excel spreadsheet (Microsoft Corp., Redmond, WA). Data were extracted independently by two authors and a third author checked a random sample of 10% of all the entries. Any differences were resolved by discussion until agreement was reached.

The quality assessment (phase II) and data extraction (phase III) for each document were carried out by two reviewers (except for Chinese articles) one of whom was a senior researcher (HC or CB). All documents published in French, Portuguese or Spanish were reviewed by those who could read those languages (PN and HC). All documents published in Chinese were reviewed by a Chinese collaborator (Y-JQ). No articles in German, Romanian or Italian were identified. In addition, a random sample of 10% of all English documents was reviewed by all reviewers. Disagreements were resolved through discussion in a meeting with all the reviewers. Data were entered into standardized electronic forms of the data extraction tool which was developed in Access (Microsoft Corp., Redmond, WA) specifically for this review (available from the authors on request). Data were collected on the study characteristics (design, geographic location, period and duration of the study), participant selection, case definitions, and ascertainment of outcome. For documents that could potentially be included in the review but had incomplete or missing data, the authors were contacted at least twice for clarification and/or additional information.

### Quantitative data synthesis and statistical analysis

The number of documents included in each phase of the systematic review was first plotted geographically using ArcView GIS software (ESRI; Redlands, CA). Results from studies reporting separately the overall prevalence or incidence rate of NCC in the population are reported for each study.

Measures of frequency: We report the proportion of patients with NCC in studies where a specific group of patients seeking care in a hospital or clinic were included. This is obtained by dividing the number of people with lesions of NCC by the number of people included in the study (all with neuro-imaging). We report the proportion of NCC among PWE using the same approach. The term prevalence is applied to the proportion of people with lesions of NCC at the CT-scan in studies where the general population or community residents without epilepsy were randomly sampled. The 95% Confidence Intervals (95% CI) of those proportions was estimating using the Clopper-Pearson exact interval provided in the Stata (StataCorp, College Station, TX) software. The annual incidence rate of hospitalized cases of NCC was calculated by dividing the number of cases discharged with an International Classification of Disease (ICD) code for cysticercosis by the person-years living in the U.S. state where the study was conducted (based on census data). The prevalence of NCC-associated epilepsy was estimated by multiplying the prevalence of epilepsy by the proportion of NCC conditional on having epilepsy in community-based studies where both these estimates were available. This prevalence was estimated using WinBugs 1.4.3© and represents the proportion of people in a community estimated to have both epilepsy and NCC.

A random-effects model was used to summarize the data on the proportion of NCC among PWE using R META package (Version 0.8–2; by Guido Schwarzer in the R-META metagen function) and the METFOR package (Version 1.3-0; by Wolfgang Viechtbauer) from R statistical software (R Development Core Team, www.R-project.org). We used the inverse variance method [15] to pool the proportion estimates in the random-effect model and calculate the appropriate 95% Confidence Interval (95% CI) [16]. Tests of homogeneity were used to determine whether it was appropriate to combine different proportions across studies, and the I^2^ index was used to summarize the total variability in proportion estimates due to between-study variation [17]. In order to determine the influence of potential outlying effect-size estimates, a sensitivity analysis was done by estimating the pooled prevalence proportion after omitting one study at a time. A mixed-effects regression model was used to determine whether the study setting (community-based or clinical-based) significantly influenced the estimated percentage of NCC among PWE value.

## Results

### Flow of included studies

**Figure 2:**
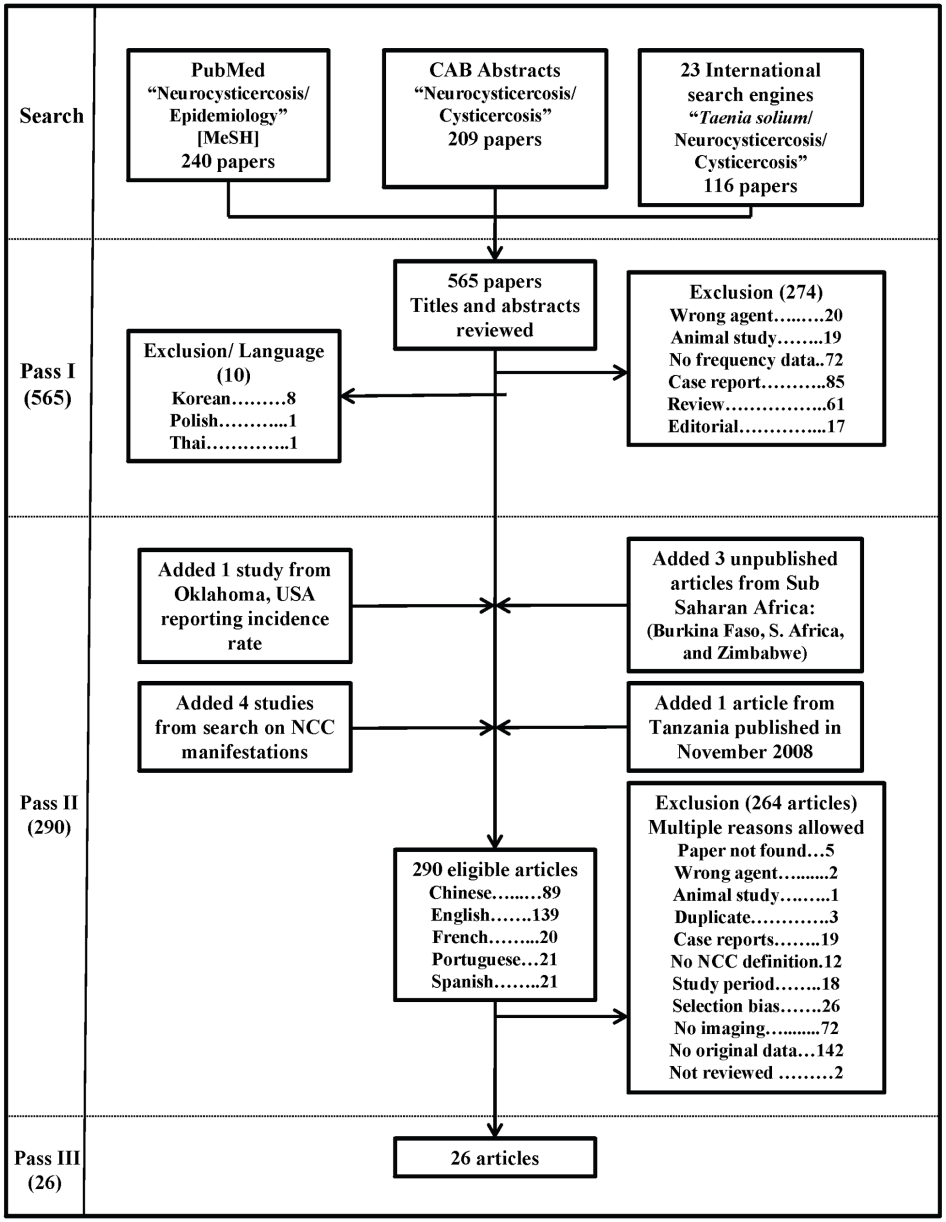
Flowchart describing the number of papers remaining at different phases of the study.

The literature search identified 565 documents that could potentially have original data on the frequency of NCC. The flow diagram in Figure 2 shows the number of papers identified in each database and the review process from phase I to phase III. Figure 2 also includes the number and reason for exclusion of documents at each phase of the review. After the first screening (phase I), 290 publications, including 9 additional studies not originally identified, were read and critically reviewed. Of the 264 articles excluded during phase II, the two most common reasons for exclusion were the lack of frequency data and the lack of neuro-imaging. Phase III included 26 documents (4.5%) containing estimates of NCC prevalence proportion or incidence rate (Table 2).

**Table 2:** Descriptive summary of the studies retained for the quantitative sorted by region.

Country, year(s) of study	Reference (language)	Design	Source population	Target population	Study population	NCC diagnosis/definition	Definition of seizures/epilepsy	% Active Epilepsy (AE)	Measure of frequency	Sampling
**Brazil, 1970–03**	[23] (English)	Cross sectional	Autopsies at the School Hospital Uberaba, Minas Gerais	2218 autopsies	2218	Autopsy/presence of cysts at histological or macroscopic examination of the brain	NA*	NA*	Proportion of NCC among all autopsies	Census
**Brazil, 1974–97**	[22] (Portuguese)	Cross sectional	Autopsies at the School Hospital Uberaba, Minas Gerais	1884 autopsies	1596 with complete data	Autopsy/not mentioned	NA*	NA*	Proportion of autopsies	Census
**Brazil, 1992–97**	[7] (Portuguese)	Cross sectional	Autopsies at the Hospital das Clinicas da Faculdade de Medicina de Ribieirao Preto-USP	2522 autopsies	2522	Autopsy/presence of cysts at histological or macroscopic examination of the brain	NA*	NA*	Proportion of NCC among all autopsies	Census
**Brazil, 1992–02**	[32] (Portuguese)	Case series	People attending the Imaging Diagnostic Center and the General Hospital of Nova Iguaçu, Rio de Janeiro	36379 CT scans of patients	36379	Brain CT-scan/acute (visualization of cysts at different development levels, but not calcified) or chronic (calcifications or granulomas as described by [57]	NA*	NA*	Proportion among patients with a CT-scan	Census
**Brazil, 1978–90**	[11] (Portuguese)	Case series	People attending the Centro de Tomografia Computadorizada e Ressonância Magnética (CETAC), Curitiba, Paraná	1000 consecutive CT scans of PWE^§^	1000	Brain CT-scan/Definite (cystic or racemose lesions), suggestive (focal intraparenchimal calcified lesions)	Clinical diagnosis. Seizures classified according to [58]	NA*	Proportion among PWE who underwent a CT scan of the brain	Census
**Bolivia, 1994**	[25] (English)	Cross sectional	9955 people screened in rural communities, Santa Cruz Department, Cordillera province	124 PWE^§^ in community	105 accepting the CT	Brain CT-scan/Lesions described in [39]	≥2 unprovoked epileptic seizures occurring >24 hours apart [59]	Not reported	Prevalence^ð^/ Proportion among PWE^§^	Community cluster/Door to door survey

Country, year(s) of study	First author (language)	Design	Source population	Target population	Study population	NCC diagnosis/definition	Definition of seizures and epilepsy	Type of epilepsy	Measure of frequency	Sampling
**Colombia, 1995**	[29] (English)	Case series	People attending 9 outpatient clinics, Instituto Neurologico de Antioquia, Medellin	643 patients with epilepsy	546 accepting the CT (≥11 years old)	Brain CT-scan/≥1 living or degenerating cyst lesions (with or without scolex) or ≥2 nodular or annular calcifications in areas unusual for physiologic calcifications	≥2 unprovoked epileptic seizures [59]	Not reported	Proportion of NCC among PWE^§^	Census
**Ecuador, 1994**	[8] (English)	Cross sectional	2327 screened residents of San Pablo del Lago	31 PWE^§^ in community	26 accepting the CT	Brain CT-scan/Based on the literature from Latin America	Based on [59]	AE^¶^ (100% defined as 5 years)	Proportion of NCC among PWE^§^	Door to door survey
**Ecuador, 2003**	[24] (English)	Cross sectional	2415 screened residents from a community in Atahualpa, 2003	24 PWE^§^ and 3 with single seizures in community	19 accepting the CT	Brain CT-scan/Lesions described in [39]	≥2 unprovoked epileptic seizures [59]	AE^¶^ (75%, defined as 5 years)	Prevalence^ð^/ Proportion among PWE^§^	Census/Door to door survey
**Honduras, 1997**	[26] (English)	Cross-sectional	6473 screened residents from Salamá rural county (11 days)	100 PWE^§^ (AE^¶^) in community	90 accepting the CT	Brain CT-scan/Modified version of lesions described in [39]	Definition from [58]–[59], [60]	AE^¶^ (100%, defined as 5 years)	Prevalence^ð^/ Proportion among PWE^§^	Census/Door to door survey
**Honduras, 1996–97**	[18] (English)	Cross-sectional	Residents of Salama rural county	80 residents with EITB+	74 accepting the CT	Brain CT-scan/Lesions compatible & pathognomonic (scolex); compatible & non pathognomonic (annular, ring-enhancing, arachnoiditis), compatible parenchymal calcifications [61]	Classified according to [58], [60]	NA*	Prevalence among seropositives	Household-cluster sampling
**Honduras, 1996–97**	[18] (English)	Cross-sectional	Residents of Salama rural county	74 residents EITB– age-sex-village matched on EITB+	74 accepting the CT	Brain CT-scan/same as above	NA*	NA*	Prevalence among sero-negatives	Household-cluster sampling
**Mexico, 1993–96**	[21] (English)	Cross-sectional	Autopsies at the General Hospital of Mexico	1274 autopsies	1274	Autopsy/pathological analysis of the brain	NA*	NA*	Proportion of NCC among all autopsies	Census

Country, year(s) of study	First author (language)	Design	Source population	Target population	Study population	NCC diagnosis/definition	Definition of seizures and epilepsy	Type of epilepsy	Measure of frequency	Sampling
**Mexico, 1999–00**	[19] (English)	Cross sectional	Residents of the rural community of Tepetzezintla	155 residents	154 accepting the CT	Brain CT-scan/Cysts and/or rounded hyperdense lesion(s) compatible with nodular brain cysticercus calcification	NA*	NA*	Prevalence	SRS^**^
**Peru, 1999–00**	[27] (English)	Cross sectional	903 permanent residents screened from the community of Tumbes River Valley	43 PWS^¥^ in community	39 accepting the CT-scan	Brain CT-scan/Lesions described in [62]	≥2 afebrile epileptic seizures separated by >24 hours [58], [60]	Epilepsy and seizures (35% AE^¶^ defined as 5 years; 30% Single seizure)	Proportion among PWS^¥^	Census/Door to door survey
**Peru, 1998–99**	[31] (Spanish)	Case series	Children consulting the neurology outpatient or emergency room at the Referral hospital Nacional Cayetano Heredia, Lima	54 (2–14 years old) with partial seizures with unknown cause of seizures	54	Brain CT/criteria from [39]	Clinical diagnosis of afebrile seizures classified according to [58]	NA*	Proportion o those with partial seizures	Census
**India, 2000–03**	[42] (English)	Case series	People attending the Institute of Neurological Sciences, Dispur, India; 2000–03	62 patients with JE	62	Brain CT-scan and MRI/Lesions described in [63]	NA*	NA*	Proportion of those with a diagnosis of JE	Census
**India, 2003–04**	[28] (English)	Cross sectional	38105 screened residents of a rural community (Kaniyambadi block), Vellore District, 2003–04	116 PWE^§^ (AE^¶^) in community	101 acceptingthe CT	Brain CT-scan/Lesions from [39] or punctuate/discrete calcifications in cerebral cortex or solitary enhancing lesions conforming with [47]	≥2 afebrile epileptic seizures	AE^¶^ (100%, defined as 5 years)	Prevalence^ð^/ Proportion among PWE^§^	Cluster sampling/door-to-door survey

Country, year(s) of study	First author (language)	Design	Source population	Target population	Study population	Neuro-imaging Investigation	Definition of seizures and epilepsy	Type of epilepsy	Measure of frequency	Sampling
**India, 2003–04**	[28] (English)	Cross sectional	12512 screened residents of an urban community, Vellore, 2003–04	78 PWE^§^ (AE^¶^) in community	61 accepting the CT	Brain CT-scan/same as above	Same as above	AE^¶^ (100%, defined as 5 years)	Prevalence^ð^/ Proportion among PWE^§^	Cluster sampling/door-to-door survey
**India, 1995–01**	[12] (English)	Case series	People attending 2 outpatient services, neurology unit, Dayanand Medical College, Ludhiana, Punjab, 1995–01	1014 patients with single seizures or recurrent seizures or epilepsy	936 with CT-scan (>12 years old)	Brain CT-scan or MRI/Presumptive NCC if 1) single or multiple cystic lesions, 2) single or multiple ring or disk-enhancing lesions, 3) single or multiple calcified nodules at the cortical-subcortical interface [64]. Criteria for solitary cysticercus granuloma used [63]	All cases defined and classified according to [59]	Single seizures (32%), recurrent seizures (19%), epilepsy (49%)	Proportion of NCC among PWS^¥^	Census
**India, 1992–93**	[13] (English)	Case Series	People attending the National Institute of Mental Health and Neuro Sciences, Bangalore, 1992–93	198 pediatric patients with partial seizures	198	Brain CT-scan/Multiple rings or disc lesions, nonenhancing focal hypodensities, eccentric scolex and calcified lesions	Simple partial seizures according to [58]	NA*	Proportion of NCC among patients with Simple Partial Seizures	Census
**India, 1995–99**	[14] (English)	Case series	Pediatric outpatients attending the Jawaharlal Institute of Postgraduate Medical Education and Research, Pondicherry, 1995–9	150 pediatric patients with partial seizures associated with motor phenomena	150	Brain CT-scan/Criteria from [39]	NA*	NA*	Proportion of NCC among children with partial seizures	Census
**Tanzania, 2006**	[10] (English)	Case series	Patients attending a rural hospital, Mbulu District, Tanzania; 2006 (7 mo)	219 patients with epilepsy	212	brain CT/lesions described in [39], [65]	≥2 afebrile seizures unrelated to acute metabolic disorders or withdrawal of drugs or alcohol [66]	AE^¶^ (98%, defined as 5 years	Proportion among PWE	Non Probability

Country, year(s) of study	First author (language)	Design	Source population	Target population	Study population	Neuro-imaging Investigation	Definition of seizures and epilepsy	Type of epilepsy	Measure of frequency	Sampling
**South Africa, 2004–05**	[30] (English)	Case series	Patients attending a rural hospital, Eastern Cape Province, South Africa 2004–05	281 patients with epilepsy	92 randomly selected for CT	Brain CT-scan/Visible cysticerci, colloidal cysts or presence of cerebral calcifications with specific CT diagnosis of NCC	≥2 unprovoked seizures >24 hours apart	AE^¶^ (96%, defined as 1 year)	Proportion among PWE^§^	SRS^**^
**Burkina Faso, 2007**	Carabin; Unpublished data (English)	Cross sectional	888 screened residents of 3 villages and self-identified PWE, Burkina Faso; 2007 (6 months)	80 PWE^§^ in community	76 accepting the CT	Brain CT-scan/lesions described in [39], [47], [65]	≥2 unprovoked seizures of CNS origin, occurring >24 hours apart, confirmed by the study physician [59]	AE^¶^ (87%, defined as 3 years)	Prevalence/ Proportion among PWE^§^	Cluster sampling and self-identified PWE^§^
**USA, 1996–98**	[33] (English)	Case series	Patients with known or suspected seizure at one of 11 hospital emergency rooms (*EMERGE*ncy ID NET), 1996–98	Patients with suspected or known seizures admitted to emergency rooms	1801 (>5 years old)	Brain CT-scan or MRI/CT scan finding(s) characteristic of NCC (i.e., multiple calcifications or multiple cystic lesions) with or without a positive serologic test, or CT scan finding(s) consistent with NCC (i.e., a single cystic, calcified, or hypodense lesion) [67]	Unspecified clinical diagnosis in emergency rooms	NA*	Proportion among seizure patients seen in Emergency rooms	Census
**USA, 1995–00**	[20] (English)	Cohort	Residents of Oregon with access to health care in 18 hospitals in 10 counties, 1995–00	Population of Oregon (n = 3,417,000)	76	Hospital discharge database/ICD-9 123.1	NA*	NA*	Incidence rate of hospitalized patients	Census/ Hospital discharge records
**USA, 2002–05**	Thompson J; unpublished data (English)	Cohort	Residents of Oklahoma with access to health care, 2002–05	Population of Oklahoma (n = 3,450,654)	40	Hospital discharge database/ICD-9 123.1/123.3	NA*	NA*	Incidence rate of hospitalized patients	Census/Hospital discharge records

NA*: Not Applicable; SRS^**^: Simple RandomSample; PWE^§^: People with epilepsy; Prevalence^ð^: Prevalence of NCC associated-epilepsy in the community; AE^¶^: Active Epilepsy; PWS^¥^: People With Seizures.

### Geographical distribution of publications on the prevalence of NCC

**Figure 3:**
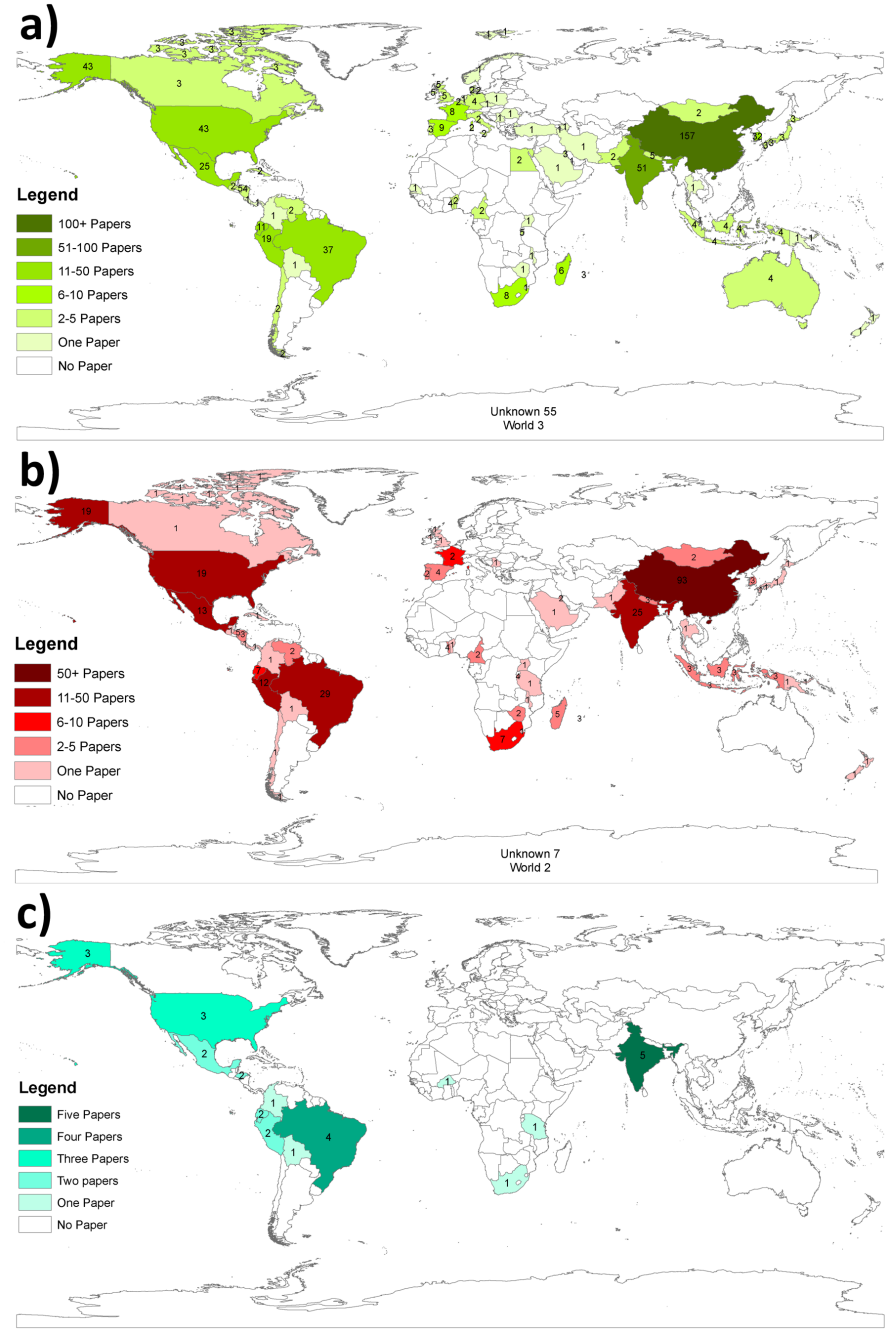
Distribution of documents identified during a systematic search of the literature from 1990 to 2008 on the frequency of neurocysticercosis which were included in the three phases of the review. a) Phase I (n = 565), b) Phase II (n = 290) and c) Phase III (n = 26).

As shown in Figures 3a–b, most of the articles identified in the search were from China, India, Brazil and the United States of America. The 26 documents that were retained for the quantitative appraisal (phase III) were from studies conducted in the WHO regions of Latin America (15), North America (3), Africa (3) and Asia (5). Figure 3c illustrates the geographic distribution of the papers that were retained for the quantitative synthesis.

### Study characteristics

Study design, the target populations and the quantity measured varied greatly across articles (Table 2). Only two studies did not sample from a target population of people with a specific symptom or disease [18]–[19]. Most studies reported the proportion of people with NCC among symptomatic target populations. Two studies from the United States of America reported the incidence rate of NCC based on hospital discharge data ([20], Thompson J, unpublished data), and four studies reported the proportion of NCC among people who were autopsied after death for any reason [7], [21]–[23]. In seven community-based studies, NCC was assessed among PWE or people with seizure disorders ([8], [24]–[28], Carabin et al, unpublished data). In another five studies, NCC was assessed among PWE attending a health clinic [10]–[12], [29]–[30]. In addition, in three studies, NCC was specifically measured among children with partial seizures [13]–[14], [31].

### Prevalence of NCC in the general population

Only one study reported the prevalence of NCC in a random sample of the general population (Table 3). In this Mexican study, 154 residents were sampled at random to receive a CT-scan of the brain [19]. The prevalence of NCC was estimated to be 9.1% (95% CI: 5.1%–14.8%). None of the sampled subjects had clinically apparent manifestations of NCC. The prevalence of NCC was considerably higher among children (aged 0–19 years old) with an estimated prevalence of 13.2% (95% CI: 7.0%–21.9%) than among adults (20–54 years old) with an estimated prevalence of 3.2% (95% CI: 0.4%–11.0%).

**Table 3:** Frequency* of NCC (95% CI) identified in the systematic review.

Country, year(s) of study	Reference	Measure of frequency	% NCC	95% CI
**Brazil, 1970–03**	[23]	Percentage of NCC among autopsies of adults	2.4	1.8–3.0
**Brazil, 1974–97**	[22]	Percentage of NCC among autopsies of adults	2.6	1.8–3.4
**Brazil, 1992–97**	[7]	Percentage of NCC among autopsies of adults	1.5	1.0–2.0
**Mexico, 1993–96**	[21]	Percentage of NCC among autopsies of adults	1.9	1.2–2.8
**India, 2000–03**	[42]	Percentage of NCC among people with JE*	19.4	14.6–24.8
**Brazil, 1992– 02**	[32]	Percentage of NCC among people with a brain CT-scan	0.2	0.15–0.24
**Peru, 1998–99**	[31]	Percentage of children (ages 2–14) with partial seizures	52.0	38.5–65.2
**India, 1995–99**	[14]	Percentage of children (ages 1–12) with partial motor seizures	2.0§	0.4–5.7
**India, 1992–93**	[13]	Percentage of children (ages 0–15) with simple partial seizures	10.1§	6.3–15.2
**USA, 1996–98**	[33]	Percentage of people with seizures seen in emergency rooms	2.1	1.5–2.9
**Honduras, 1995**	[18]	Prevalence among people EITB positive	23.0	14.0–34.2
**Honduras, 1995**	[18]	Prevalence among people EITB negative age-sex-village matched to EITB+	18.9	10.7–29.7
**Peru, 1999–00**	[27]	Prevalence among people EITB positive and without epilepsy	34.0	21.5–48.3
**Peru, 1999–88**	[27]	Prevalence among a SRS** of people EITB negative and without epilepsy	13.8	6.1–25.4
**Mexico, 1999–00**	[19]	Prevalence among a SRS** of people in the community	9.1	5.1–14.8
**Ecuador, 1994**	[8]	Percentage among a SRS** from people without epilepsy in a community	14.4	8.5–21.2
**Ecuador, 2003**	[35]	Percentage among a age-gender matched (to epilepsy cases) sample of people without epilepsy in a community	5.2	0.1–26.0
**USA, 1995–00**	[20]	Incidence rate of inpatients with NCC (/10^5^ person-years)	1.5	
USA, 2002–05	Thompson, unpublished	Incidence rate of inpatients with NCC (/10^5^ person-years)	0.29	

*JE: Japanese encephalitis;

**SRS: Simple random sample;

**§:** NCC excluded patient with solitary cyst or granuloma in the brain.

### Prevalence of NCC among people without epilepsy and epileptic seizures

Two studies were conducted among patients without epilepsy and epileptic seizures, both part of a larger door-to-door survey to identify people with epilepsy conducted in Ecuador. In the first study, lesions suggestive of NCC at CT were found in 17 out of 118 randomly selected people without epilepsy [8], for a percentage of 14.4% (95%CI: 8.5%–21.2%). In the second study, NCC lesions were identified among a matched age-gender sample of 19 people without epilepsy (matched to those with epilepsy), for a percentage of 5.2% (95%CI: 0.1%–26.0%) [24]. No details on the age distribution or types of lesions found were provided in those articles.

### Proportion of NCC among community residents seropositive and seronegative to the presence of antibodies to *T. solium*

The proportion of NCC in seropositive and seronegative community residents was estimated in two studies [18], [27]. In Honduras, 480 people aged 2 years and older from Salama county, provided a blood sample to estimate the seroprevalence of cysticercosis using a Western Blot (EITB) [18]. A total of 80 people tested positive to the EITB, of whom 74 accepted to receive a CT-scan of the brain. An age-gender-village matched sample of 74 sero-negative people also received a CT-scan of the brain. In the second study, 825 out of 913 residents of seven villages of the district of Matapalo in Peru provided a blood sample for EITB testing. A random sample of 53 of 60 people testing positive and 58 of 60 people testing negative to EITB without epilepsy accepted to have a CT-scan of the brain [27]. The percentage of NCC was 23.0% (95% CI: 14.0%–34.2%) and 34.0% (95% CI: 21.5%–48.3%) among seropositive participants, and 18.9% (95% CI: 10.7%–29.7%) and 13.8% (95% CI: 6.1%–25.4%) among seronegative patients in Honduras and Peru, respectively. Age-stratified prevalence of NCC results was not reported. It is important to note that none of those groups represents the general population of those villages.

### Estimates of annual incidence rate of hospitalized NCC cases

Two studies, both using data from discharge diagnosis of patients hospitalized in the United States of America, reported estimates of the incidence rate of hospitalized NCC per 100,000 person-years (Table 2). In Oregon and Oklahoma, the incidence rates were estimated at 1.50 per 100,000 person-years and 0.29 per 100,000 person-years, respectively ([20], Thompson, unpublished data).

### Proportion of NCC in selected groups of clinical patients

As expected, the proportion of NCC was extremely variable among studies with different source and target populations (Table 3). In Peru, the percentage of NCC in children with partial seizures was 52.0% (95% CI: 38.5%–65.2%) [31]. This estimate was considerably lower in two studies conducted among children with partial seizures in India (Table 2) [13]–[14]. The latter two studies did not consider solitary calcified cysts as NCC lesions.

In a study conducted among a group of patients attending two imaging diagnostic centers in Brazil, the percentage of NCC was estimated at 0.20% (95% CI: 0.15%–0.24%) [32].

We found only one study from a developed country (United States) reporting the percentage of NCC among people with seizures attending emergency rooms [33]. In that study, the overall prevalence of NCC was 2.1% (95% CI: 1.5%–2.9%), but was 9.1% (95% CI: 6.2%–12.8%) among Hispanics.

### Proportion of NCC among autopsied patients

Four studies reported the proportion of NCC among people who were autopsied [7], [21]–[23]. The percentages were similar across the four studies, varying from 1.5% to 2.6%, with three of the four studies conducted in the same area of Brazil (Table 3).

### Prevalence of NCC-associated epilepsy in community-based studies

The estimated prevalence of people with NCC-associated epilepsy in community-based studies ranged from 0.1% in India [28] to 1.3% in a study of three rural communities in Burkina Faso (Carabin, unpublished data) (Table 4). Such estimates could not be combined because the proportion of NCC was never obtained from all PWE in the population.

**Table 4:** Prevalence (%) of NCC-associated epilepsy and 95% Bayesian Credible Interval (95% BCI) in community-based studies.

Country, year	Reference	Number of people with NCC (number with CT-scans)	Number of people with Epilepsy	Number of people screened for the presence of epilepsy	% NCC-associated epilepsy in the study population (95%BCI)
Bolivia, 1994	[25]	29 (105)	124	9955	0.35 (0.24–0.49)
Ecuador, 1994	[8] *	14 (26)	31	2723	0.62 (0.36–0.97)
Ecuador, 2003	[24]	5 (19)	23	2415	0.27 (0.11–0.54)
Honduras, 1997	[26] *	33 (90)	100	6473	0.57 (0.40–0.78)
Burkina Faso, 2007	Carabin; unpublished	17 (68)	39	888	1.32 (0.81–2.05)
Urban India, 2003	[28] *	35 (101)	116	38105	0.11 (0.07–0.15)
Rural India, 2003	[28] *	11 (61)	78	12512	0.12 (0.06–0.20)

*These studies only included patients with active epilepsy (defined by at least one epileptic seizure in the past 5 years).

### Proportion of NCC among people with epilepsy

**Figure 4:**
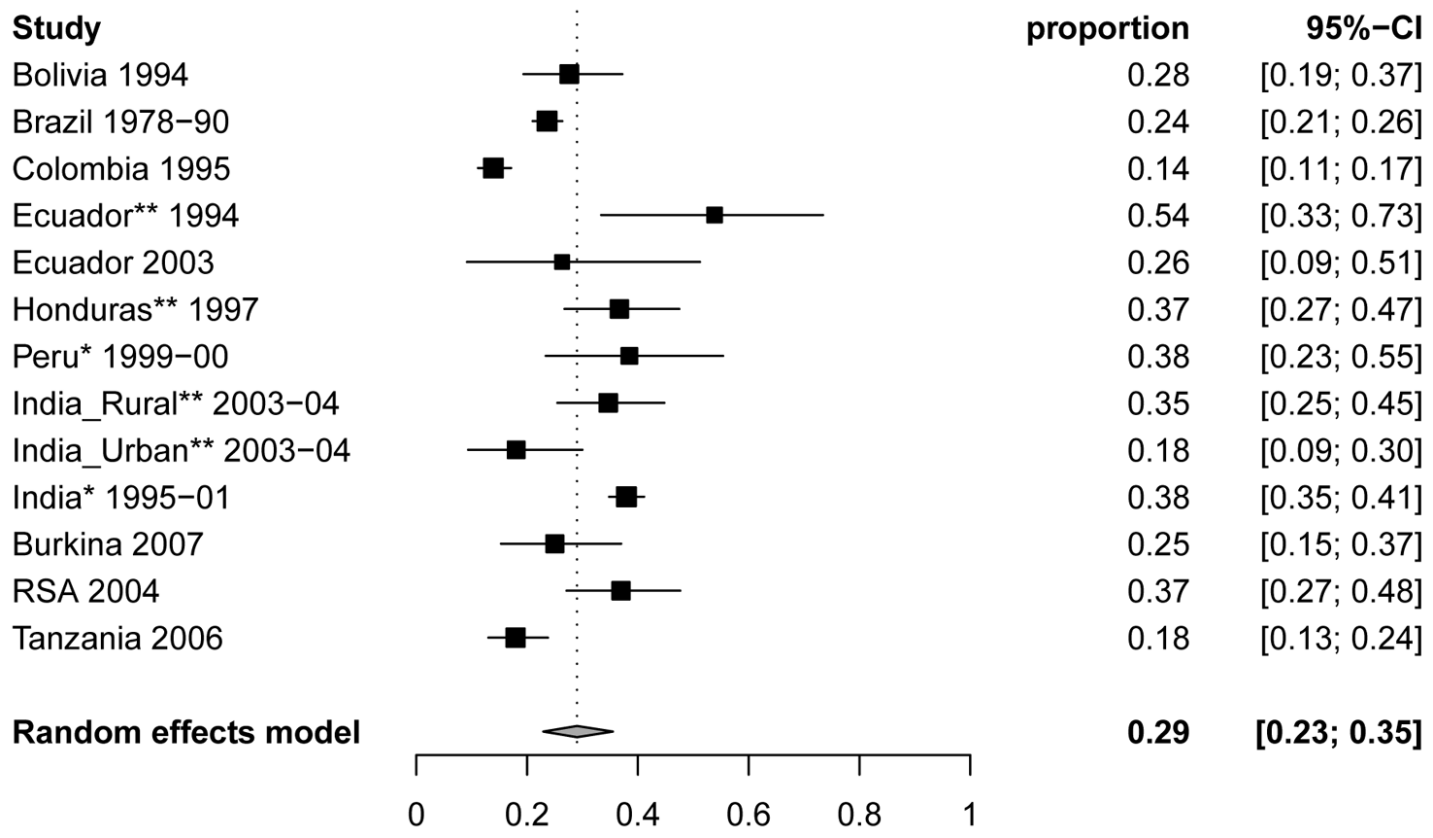
Forest plots of the proportion of NCC (95% CI) in people with epilepsy from 12 studies reporting from cases in all age groups. *Indicates studies among people with epilepsy and seizures. ** Indicates studies among people with active epilepsy only.

The proportion of NCC among PWE was remarkably homogeneous across studies conducted in children and adults (Figure 4). The lowest estimated percentage was from a study conducted in several urban clinics in Colombia, with an estimated 13.9% NCC among PWE (95% CI: 11.1%–17.1%) [29]. Interestingly, all patients with single calcifications in that study were considered as negative for NCC. The pooled estimate across 12 studies from the random-effects model for the percentage of NCC among PWE of all ages was 29.0% (95% CI: 22.9%–35.5%). The I^2^ statistic indicated that 92.5% (95% CI: 88.1%–94.6%) of the total variability in the percentage values was due to between-study variation. No study had a significant impact on the result and the between-study variability was not explained by a single study.

We ran the model excluding the study from Peru where some single seizures cases were included [27] and only including patients with epilepsy or recurring acute symptomatic seizures from one of the India studies [12]. The resulting pooled estimate is 27.6% (95%CI: 22.8%–32.6%), which is very close to the previous estimate.

We also ran random-effect models stratified by the target population (clinical vs community). The estimates were 31.7% (95% CI: 25.6%–38.2%) for community-based and 25.4% (95%CI: 16.3%–35.7%) for clinical-based studies. A mixed-effects regression model was used to determine whether the study setting (community-based or clinical-based) significantly influenced the percentage value. The estimated percentage among clinic-based studies is expected to be 5.9% lower (absolute difference) than that for community-based studies (95% CI: 17.2% lower to 5.5% higher), which is not statistically significant (p = 0.31).

**Figure 5:**
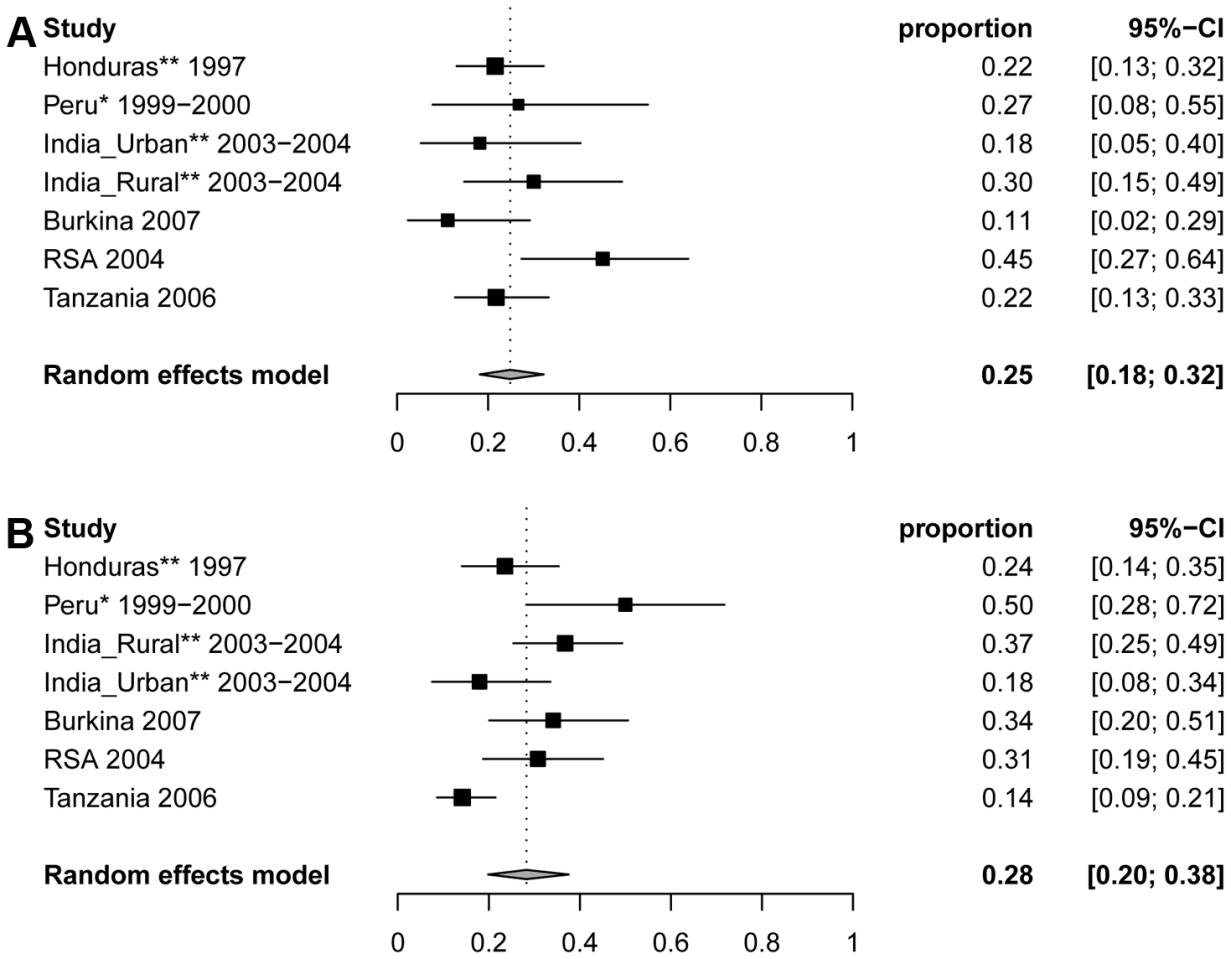
Forest plots of the proportion of NCC (95% CI) among people with epilepsy in children and adults. a) people aged between 0 and 19 years old and b) aged 20 years old or more. *Indicates studies among people with epilepsy and seizures. ** Indicates studies among people with active epilepsy only.

Only five of the 12 studies had sufficient information to obtain estimates stratified by two broad and consistent age groups. In patients less than 20 years of age, the percentage of NCC among PWE ranged from 11.1% (95% CI: 2.4%–29.2%) in a community-based study in Burkina Faso (Carabin, unpublished data) to 45.2% (95% CI: 27.3%–64.0%) in the Eastern Cape Province of South Africa [30], with an overall estimate of 24.8% (95% CI: 18.2%–32.2%) (Figure 5a). The I^2^ statistic suggested that 43.1% (95%: CI 0%–76.1%) of the total variability in the values was due to between-study variation. In the analysis of adults aged 20 to 54 years, the estimate of the percentage of NCC among PWE was more variable, ranging from 14.2% (95% CI: 8.6%–21.5%) in an outpatient clinic in Tanzania [10] to 50.0% (95% CI: 28.2%–71.8%) in a community in Peru [27], with an overall estimate of 28.3% (95% CI: 19.9%–37.5%) (Figure 5b). I^2^, the percentage of total variation across studies due to between-study heterogeneity, was 74.8% (95% CI: 46.6%–88.2%).

## Discussion

This is the first study to systematically collect data on the frequency of NCC worldwide Our results demonstrate that there is not sufficient evidence at this time to estimate the prevalence of NCC globally. However, our study is the first meta-analysis to summarize the proportion of NCC among PWE, and suggest that nearly one-third of PWE living in endemic communities show lesions of NCC in their brain. Our study is particularly comprehensive because it used 25 different databases to identify published information and included documents published in four different languages.

### Methodological issues

After a systematic review, only studies that are likely to be valid and are similar enough in their methods and definitions should be reported [34]. Only 4.5% of all publications identified were considered valid enough to be included in the systematic review. However, only studies reporting on the proportion of NCC among PWE were similar enough to be combined in a pooled estimate.

The second most common reason for exclusion of documents, after an absence of measurement of NCC frequency, was the lack of neuro-imaging for the diagnosis of NCC. Underdeveloped countries where sanitation and proper pig management methods are lacking are often endemic for *T. solium* infections [8], [35]. These same countries are those where imaging facilities are scarce [28]; this especially applies to Sub-Saharan Africa [36]. The absence of appropriate diagnostic technologies leads to an unequal distribution of studies included in this systematic review. Indeed, there are few articles from Africa, the Western Pacific, the Eastern part of Europe and Asia, with the exception of India and China. The small number of studies from the Middle East and parts of Africa is not surprising since pig rearing and pork consumption are rare in those areas. . Yet, a small number of studies have reported NCC cases in Islamic or Jewish communities [37] and it would be commendable to conduct more studies of NCC in those communities. Infection usually occurs when individuals from endemic areas are taeniasis carriers [38]. Restriction to studies using neuroimaging was based on the internationally accepted descriptions of lesions of NCC which requires the use of imaging for the diagnosis of this disease [39].

The third most common reason for exclusion of a study was the use of flawed methodology. Studies were excluded that did not mention when and where they were conducted as were primary studies with a high potential of selection bias, since the strength of a systematic review depends on the quality of the primary studies that are included [40]. Methodologically poor studies may bias the conclusions and produce incorrect overall estimates when quantitative methods are used [41].

### The proportion of NCC in specific populations

Target and study populations were very different across the documents we analyzed. For example, studies were conducted on patients with Japanese encephalitis (JE) [42], a simple random sample of people living in a community in Mexico [19], patients attending imaging diagnostic centers [11], [32] and children with partial seizures [13]–[14], [31]. The frequency of NCC across these populations varied widely and the heterogeneity between studies hindered the calculation of an overall estimate. Only studies conducted among PWE were homogeneous enough to warrant the use of a meta-analysis.

The only cross-sectional study among a random sample of the population found a prevalence of NCC lesions, all calcified, of 9.1% [19]. This prevalence is similar to what was found in one study conducted in Ecuador where people without epilepsy and epileptic seizures were selected at random [8]. None of those studies reported on the presence of other possible past or present neurological manifestations of NCC. Therefore, even though it is possible that those participants were truly asymptomatic, it is impossible to know with certainly in the absence of a full neurological examination and anamnesis.

In another study, people who were sero-positive to the EITB and an age-sex-village matched sample of sero-negative people underwent a CT-scan of the brain [18]. Even though this study is interesting in showing that the prevalence of NCC was very similar in the two groups, suggesting the poor performance of the EITB to detect NCC in community-based studies conducted in endemic areas, it cannot be used to estimate the prevalence of NCC. This is because people who are sero-positive (and their match controls) may represent people who are more exposed to the larval stages of *T. solium* in their community. Given that the incubation of NCC is unknown, those participants testing negative to the EITB may have been exposed a long time ago but have seen their immunity wane with time. Those cases may also have never developed antibodies to the brain infection. Such studies are unlikely to be repeated in the future. Due to potential adverse effects of the contrast materials used for CT or MRI, it is usually considered unethical to perform neuro-imaging in apparently healthy individuals. This limits our ability to truly measure the burden of NCC as some people may be asymptomatic for extended periods of time [43]. The study among participants seropositive and seronegative to EITB from Peru is even more difficult to interpret since it was conducted only among those without epilepsy [27].

We identified four studies conducted in autopsied patients from large hospitals in Brazil and Mexico [7], [21]–[23]. These results were very similar, but three of the four studies were conducted in the same province of Brazil, thus representing the same population. In addition, an extrapolation of those results to the general population is impossible since people who are autopsied are likely to systematically differ from the general population.

### The prevalence of NCC-associated epilepsy in community-based studies

The prevalence of people with NCC-associated epilepsy in community-based studies varied considerably. This prevalence is the product of the prevalence of PWE in the community and the proportion of NCC among PWE. Since the prevalence of NCC among PWE tends to be similar across studies (about one-third), the prevalence of epilepsy in communities is the parameter that contributes the most to the observed variability across communities. There are diverse, competing etiologies for epilepsy across countries and in addition to NCC include malaria, paragonomiasis, toxocariasis, and other parasites of the brain [5], a plethora of metabolic disorders, traumatic brain injuries as well as febrile seizures during childhood [44]. The inconsistency in prevalence estimates can also be explained by the fact that the definition of epilepsy and of active epilepsy varied from study to study. Some authors used a cutoff of one year of unprovoked seizures whereas others used three or five years to define active epilepsy.

### The proportion of NCC among PWE

The results from our meta-analysis show that epilepsy is consistently associated with NCC in over one quarter of patients residing in endemic regions. This result was very robust, regardless of the type of epilepsy, if single epileptic seizures were included or not, and where and among whom the study was conducted. In an older study of 100 consecutive patients with epilepsy, Medina et al. found a prevalence of 50% for NCC [45]. Another study in South Africa conducted on 578 PWE, calculated a proportion with NCC of 28% [46], which is very close to the average in the articles reviewed in this meta-analysis. These estimates confirm the importance of NCC infection in the etiology of epilepsy in developing countries [45] and suggest that NCC may be associated with a very large burden in cysticercosis endemic areas where epilepsy is prevalent.

It is difficult to determine if our finding of the proportion of NCC lesions among PWE is an over or underestimate of the truth. First, epidemiological studies are generally conducted in areas where the infection is expected to be common. Second, as describe in the Mexican study [19], some proportion of the population have NCC lesions in the brain that are not manifesting (at least at the time of the study). These two factors would support an overestimation of the proportion of epilepsy that could be attributable to NCC in endemic communities. However, in a pilot study conducted in three communities in Burkina Faso, one of the communities selected had very few pigs and most of the residents were Muslim. There were no NCC cases among PWE in that community, which was located only about 10 km from another community where about 45% of PWE had lesions of NCC (Carabin, unpublished results). The combined proportion of NCC among PWE was 29%. This suggests that our estimate may be accurate if the selected study communities represented rural areas of a country. However, if communities with clusters of NCC were specifically selected, then our results would be an overestimation of the country-wide reality.

In three of the studies, single calcifications and/or single enhancing lesions were not considered as lesions compatible with NCC. This goes against the lesions described in Del Brutto et al. [39] which consider single, calcified lesions as a minor criterion, and does not consider the fact that some single calcified lesions may very well be NCC if combined with a positive result to EITB [47]. This could lead to an underestimate of the true prevalence. Even though not all single calcified lesions of the brain will be NCC, we assessed what impact the inclusion of all of those lesions as NCC would have had on the results. In the two India studies, the percentage of NCC among children with partial seizures would have increased from 10.1% to 38.1% in one study [14] and from 2.0% to 12.0% in the other study [13]. In the study by Palacio et al. in Colombia [29], the estimate of NCC among PWE would have increased from 13.9% to 22.7%. We conducted a sensitivity analysis assuming that those solitary cysts were NCC in the Palacio study. This analysis yielded a pooled estimate of NCC among PWE of 30.3% (95% CI: 25.3%–35.5%), which is very similar to the previous estimate.

### The proportion of NCC among children with epilepsy and partial seizures

It is generally believed that NCC is a cause of late-onset epilepsy. Our meta-analysis contradicts this belief by obtaining very similar pooled estimates of the proportion of NCC among PWE in adults (aged 20 years old or more) and children (aged less than 20 years old). The proportion of NCC among children with partial seizures varied considerably. However, it does support the fact that children are affected by NCC.

### Limitations of the study

Our study has some limitations with regards to missing data, potential biases, and misclassification. Although a very broad search in seven different languages was conducted, relevant papers may have been overlooked. This situation may have the consequence of introducing a bias in the synthesis we were aiming to produce. Another potential bias may be publication bias, as we mainly considered published papers [48]. Apart from the specific region of Sub-Saharan Africa, we were unable to locate unpublished studies from other areas. To our knowledge, there have not been any published studies of NCC using neuro-imaging conducted in Viet-Nam, Cambodia, Laos or the Philippines (Willingham, personal communication, May 2009).

Another important limitation is that the ascertainment of NCC cases remains a problem. Although CT and MRI are considered the best tools to diagnose NCC, they can miss early stages of the larvae infestation in the brain [49]. The definitive diagnosis of NCC has to be made by a set of methods including neuro-imaging procedures, histological techniques and immunological investigations, because the use of any single method may provide flawed diagnoses [50]. As mentioned earlier, many neuro-imaging lesions are not pathognomonic of NCC [51]. Unequivocal diagnosis can only be achieved by absolute recognition of a scolex on neuro-imaging, or by biopsy or autopsy [39], [49]. However, invasive procedures are rarely routinely performed for diagnostic purposes [49]. Hence, our findings, which rely on a CT-scan diagnosis, may over- or underestimate the actual frequency of NCC. Whether it is an over-estimate due to counting lesions that are not NCC depends on how much of NCC does manifest as epileptic seizures. Indeed, since all our studies are cross-sectional in nature, it is impossible to determine if the NCC lesion is indeed the cause of the epileptic seizures. This problem could be exacerbated by the fact that radiologists may have had a wide variance in CT interpretation, especially for small, calcified lesions [51].

A further limitation of this systematic review is that most published studies have been based on small sample sizes. Gender and age-specific data were often not available and, when reported, the age groups were not consistent. Most of the authors we contacted for additional information did not answer our correspondence. Hence, we could only report the prevalence estimates from a sub-sample of the studies and in two very broad age groups. Interestingly, we noticed that the prevalence of NCC among PWE in children aged less than 20 was much higher in South Africa. This supports prior reports suggesting that NCC may be more common in children in South Africa than elsewhere [52]. Gender-specific estimates could not be calculated, and we could not verify whether females are more affected by NCC, as has been previously hypothesized [50].

Finally, all the available literature is based on cross-sectional studies in communities or clinics that selected PWE and offered them a CT-scan of the brain. It is impossible to determine the temporality of the link between NCC and epilepsy in such study design. Unfortunately, a cohort study of people developing brain lesions of NCC which follows them to see if they develop epilepsy is not ethically feasible. Among the literature reviewed, one was a prevalence case-control study and reported a prevalence odds ratio of 6.9 between NCC and epilepsy [8].

This systematic review has shown several challenges for the assessment of NCC's global burden. One way to improve the assessment of the global burden of NCC would be to encourage and enforce the use of a standard diagnosis for NCC, such as that developed by Del Brutto et al. [39]. This may require the provision of adequate diagnostic tools and expertise to all endemic countries. A second step would be to follow the proposal of some authors to declare NCC an international reportable disease [53]. This proposal was reviewed and rejected by the World Health Assembly in 2003 because it was felt that only diseases which can lead to large-scale international outbreaks should be included in the list of internationally notifiable diseases [54]. However, countries were encouraged to add this disease to their national list of notifiable diseases. Compulsory notification would have the benefit of providing accurate quantification of NCC prevalence in endemic areas. In 1992, the municipality of Ribeirão Preto in Brazil, decided to make NCC a reportable disease in that region [7]. With the standardization of NCC diagnostic criteria and compulsory notification, the global burden of NCC would be easier to establish. A future systematic review of published and unpublished documents, extended to all relevant documents reporting NCC cases, will help capture more complete data. In order to standardize how NCC is reported in articles, we also propose to share the Access™ data extraction tools that were developed for this review. Data collected in the same way will be easier to combine. Collaborative data would improve the focus and decision-making regarding preventive measures for a disease that has severe complications.

Despite these challenges, this study found that approximately one-third of people with epilepsy living in regions endemic for *T. solium* were associated with NCC. While this estimate may be biased due to measurement error, its robustness across populations and studies suggest that it is likely to be accurate. The number of DALYs lost due to epilepsy worldwide was estimated to be 6,223,000, with slightly higher values for males (3,301,000) than for females (2,922,000) [55]. Many risk factors for epilepsy are linked with a lower level of economic development; thus, the burden is highest in South Asia, followed by Sub-Saharan Africa [54]. In India, the DALY for epilepsy estimated in 2002 was 1539 [56].

Given the very robust estimate of the proportion of people with NCC lesions among PWE and, in some cases people with epileptic seizures, it may be possible to use the range of the percentage of NCC among PWE to estimate the number of NCC-associated epilepsy cases in endemic areas. To achieve this goal, we will need to obtain information on the prevalence of epilepsy in areas that are endemic for cysticercosis, that is, those countries were sanitation is poor, pork consumption occurs, and pigs have access to human feces. In the literature that we have reviewed, we have not come across publications suggesting that NCC is not endemic in areas where these three conditions are met. By multiplying the range of prevalence of epilepsy by the range of the proportion of NCC among PWE in each endemic area, we would obtain a range of values for the prevalence of NCC-associated epilepsy. Since epilepsy has been reported as the most common manifestation of NCC, such estimates will probably capture the majority of NCC-associated burden. This information will ultimately lead to the estimation of prevalence DALYs associated with NCC.

## Supporting Information

Checklist S1 PRISMA checklist.
(0.07 MB DOC)

Flowchart S1 PRISMA flow diagram.
(0.06 MB DOC)

## References

[1] Murray CJ, Lopez AD, Jamison DT (1994). The global burden of disease in 1990: summary results, sensitivity analysis and future directions.. Bull World Health Organ.

[2] Murray CJ, Lopez AD (1997). Global mortality, disability, and the contribution of risk factors: Global Burden of Disease Study.. Lancet.

[3] Hotez PJ, Brown SA (2009). Neglected tropical disease vaccines.. Biologicals.

[4] Edwards G, Krishna S (2004). Pharmacokinetic and pharmacodynamic issues in the treatment of parasitic infections.. Eur J Clin Microbiol Infect Dis.

[5] Hotez PJ, Brindley PJ, Bethony JM, King CH, Pearce EJ (2008). Helminth infections: the great neglected tropical diseases.. J Clin Invest.

[6] Wagner RG, Newton CR (2009). Do helminths cause epilepsy?. Parasite Immunol.

[7] Chimelli L, Lovalho AF, Takayanagui OM (1998). [Neurocysticercosis: contribution of autopsies in the consolidation of mandatory notification in Ribeirao Preto-SP,Brazil].. Arq Neuropsiquiatr.

[8] Cruz ME, Schantz PM, Cruz I, Espinosa P, Preux PM (1999). Epilepsy and neurocysticercosis in an Andean community.. Int J Epidemiol.

[9] Varma A, Gaur KJ (2002). The clinical spectrum of neurocysticercosis in the Uttaranchal region.. J Assoc Physicians India.

[10] Winkler AS, Blocher J, Auer H, Gotwald T, Matuja W (2009). Epilepsy and neurocysticercosis in rural Tanzania-An imaging study.. Epilepsia.

[11] Trentin A-P, Teive HAG, Tsubouchi MH, de Paola L, Minguetti G (2002). [Tomographic findings in 1000 consecutive patients with antecedents of epileptic seizures].. Arq Neuropsiquiatr.

[12] Singh G, Singh P, Singh I, Rani A, Kaushal S (2006). Epidemiologic classification of seizures associated with neurocysticercosis: observations from a sample of seizure disorders in neurologic care in India.. Acta Neurol Scand.

[13] Nair KP, Jayakumar PN, Taly AB, Arunodya GR, Swamy HS (1997). CT in simple partial seizures in children: a clinical computed tomography study.. Act Neurol Scand.

[14] Hussain J, Srinivasan S, Serane VT, Mahadevan S, Elangovan S (2004). Cranial computed tomography in partial motor seizures.. Indian J Pediatr.

[15] Cooper H, Hedges LV (1994). The Handbook of Research Synthesis.

[16] DerSimonian R, Laird N (1986). Meta-analysis in clinical trials.. Control Clin Trials.

[17] Higgins JP, Thompson SG (2002). Quantifying heterogeneity in a meta-analysis.. Stat Med.

[18] Sánchez AL, Lindbäck J, Schantz PM, Sone M, Sakai H (1999). A population-based, case-control study of *Taenia solium* taeniasis and cysticercosis.. Ann Trop Med Parasitol.

[19] Fleury A, Gomez T, Alvarez I, Meza D, Huerta M (2003). High prevalence of calcified silent neurocysticercosis in a rural village of Mexico.. Neuroepidemiol.

[20] Townes JM, Hoffmann CJ, Kohn MA (2004). Neurocysticercosis in Oregon, 1995–2000.. Emerg Infect Dis.

[21] Herrera LA, Benita-Bordes A, Sotelo J, Chávez L, Olvera J (1999). Possible relationship between neurocysticercosis and hematological malignancies.. Arch Med Res.

[22] Lino RS, Reis MA, Teixeira VPA (1999). [Occurrence of encephalitic and cardiac cysticercosis (Cysticercus cellulosae) in necropy.]. Rev Saude Pub.

[23] Lino RS, Faleiros AC, Vinaud MC, Oliveira FA, Guimarães JV (2007). [Anatomopathological aspects of neurocysticercosis in autopsied patients].. Arq Neuropsiquiatr.

[24] Del Brutto OH, Santibáñez R, Idrovo L, Rodrìguez S, Díaz-Calderón E (2005). Epilepsy and neurocysticercosis in Atahualpa: a door-to-door survey in rural coastal Ecuador.. Epilepsia.

[25] Nicoletti A, Bartoloni A, Sofia V, Bartalesi F, Chavez JR (2005). Epilepsy and neurocysticercosis in rural Bolivia: a population-based survey.. Epilepsia.

[26] Medina MT, Durón RM, Martínez L, Osorio JR, Estrada AL (2005). Prevalence, incidence, and etiology of epilepsies in rural Honduras: the Salama Study.. Epilepsia.

[27] Montano SM, Villaran MV, Ylquimiche L, Figueroa JJ, Rodriguez S (2005). Cysticercosis Working Group in Peru. Neurocysticercosis: association between seizures, serology, and brain CT in rural Peru.. Neurology.

[28] Rajshekhar V, Raghava MV, Prabhakaran V, Oommen A, Muliyil J (2006). Active epilepsy as an index of burden of neurocysticercosis in Vellore district, India.. Neurology.

[29] Palacio LG, Jiménez I, Garcia HH, Jiménez ME, Sánchez JL (1998). Neurocysticercosis in persons with epilepsy in Medellin, Colombia. The Neuroepidemiological Working Group of Antioquia.. Epilepsia.

[30] Foyaca-Sibat H, Cowan LD, Carabin H, Targonska I, Anwary MA (2009). Accuracy of serological testing for the diagnosis of prevalent neurocysticercosis in outpatients with epilepsy, Eastern Cape Province, South Africa.. PLoS Negl Trop Dis.

[31] Gaffo AL, Guillén-Pinto D, Campos-Olazábal P, Burneo JG (2004). [Cysticercosis as the main cause of partial seizures in children in Peru].. Rev Neurol.

[32] Mendes EC, da Silva SS, Fonseca EA, de Souza HR, de Carvalho RW (2005). [Human neurocysticercosis in Baixada Fluminense, Rio de Janeiro State, Brazil].. Arq Neuropsiquiatr.

[33] Ong S, Talan DA, Moran GJ, Mower W, Newdow M (2002). Neurocysticercosis in radiographically imaged seizure patients in U.S. emergency departments.. Emerg Infect Dis.

[34] Green S (2005). Systematic reviews and meta-analysis.. Singapore Med J.

[35] Pal DK, Carpio A, Sander JW (2000). Neurocysticercosis and epilepsy in developing countries.. J Neurol Neurosurg Psychiatry.

[36] Nguekam JP, Zoli AP, Zogo PO, Kamga AC, Speybroeck N (2003). A seroepidemiological study of human cysticercosis in West Cameroon.. Trop Med Int Health.

[37] Rousseau MC, Guillotel B, Delmont J (1999). [Neurocysticercosis in the South-East of France 1988–1998].. Presse Med.

[38] Hussein FMY, Alhajri FA, Buriki KB, El Beltaji AHJ, Ovais MI (2003). Neurocysticercosis in Kuwait: Computerized Tomography and Magnetic Resonance Imaging Findings.. Kuwait Med J.

[39] Del Brutto OH, Rajshekhar V, White AC, Tsang VC, Nash TE (2001). Proposed diagnostic criteria for neurocysticercosis.. Neurology.

[40] Simunovic N, Sprague S, Bhandari M (2009). Methodological issues in systematic reviews and meta-analyses of observational studies in orthopaedic research.. J Bone Joint Surg Am.

[41] Khan KS, Daya S, Jadad A (1996). The importance of quality of primary studies in producing unbiased systematic reviews.. Arch Intern Med.

[42] Handique SK, Das RR, Saharia B, Das P, Buragohain R (2008). Coinfection of Japanese encephalitis with neurocysticercosis: an imaging study.. Am J Neuroradiol.

[43] Nikolic S, Stevanovic G (2006). [Neurocysticercosis–pathogenesis and clinical aspects].. Srp Arh Celok Lek.

[44] Sander JW (2003). The epidemiology of epilepsy revisited.. Curr Opin Neurol.

[45] Medina MT, Rosas E, Rubio-Donnadieu F, Sotelo J (1990). Neurocysticercosis as the main cause of late-onset epilepsy in Mexico.. Arch Intern Med.

[46] Van As AD, Joubert J (1991). Neurocysticercosis in 578 black epileptic patients.. S Afr Med J.

[47] Rajshekhar V, Chandy MJ (1997). Validation of diagnostic criteria for solitary cerebral cysticercus granuloma in patients presenting with seizures.. Acta Neurol Scand.

[48] Berlin JA, Begg CB, Louis TA (1989). An assessment of publication bias using a sample of published clinical trials.. J Am Stat Assoc.

[49] Sanchez AL, Ljungstrom I, Medina MT (1999). Diagnosis of human neurocysticerocosis in endemic countries: a clinical study in Honduras.. Parasitol Int.

[50] Benedeti MR, Falavigna DL, Falavigna-Guilherme AL, Araujo SM (2007). [Epidemiological and clinical profile of neurocysticercosis patients assisted by the Hospital Universitario Regional de Maringa, Parana, Brazil].. Arq Neuropsiquiatr.

[51] Garcia HH, Herrera G, Gilman RH, Tsang VC, Pilcher JB (1994). Discrepancies between cerebral computed tomography and western blot in the diagnosis of neurocysticercosis. The Cysticercosis Working Group in Peru (Clinical Studies Coordination Board).. Am J Trop Med Hyg.

[52] Mafojane NA, Appleton CC, Krecek RC, Michael LM, Willingham AL (2003). The current status of neurocysticercosis in Eastern and Southern Africa.. Acta Trop.

[53] Román G, Sotelo J, Del Brutto O, Flisser A, Dumas M (2000). A proposal to declare neurocysticercosis an international reportable disease.. Bull World Health Organ.

[54] World Health Organization (WHO) (2003). Control of neurocysticercosis. Report of the Secretariat, Fifty-sixth World Health Assembly. Document A56/10.

[55] Mathers CD, Lopez AD, Murray CJL, Lopez AD, Mathers CD, Essati M, Jamison DT, Murray JL (2006). The Burden of Disease and Mortality by Condition: Data, Methods, and Results for 2001.. Global Burden of Disease and Risk Factors.

[56] World Health Organization (2009).

[57] Andrade-Filho AS, Souza APQU, Souza YMA (1998). Neurocisticercose: diagnóstico. Revisão. Parte II.. RBNP.

[58] Commission on Classification and Terminology of the International League Against Epilepsy (1981). Proposal for revised clinical and electroencephalographic classification of epileptic seizures.. Epilepsia.

[59] Commission on epidemiology and prognosis, International League Against Epilepsy (1993). Guidelines for epidemiologic studies of epilepsy.. Epilepsia.

[60] Commission on Classification and Terminology of the International League Against Epilepsy (1989). Proposal for revised classification of epilepsies and epileptic syndromes..

[61] Sotelo J, Guerrero V, Rubio F (1985). Neurocysticercosis: a new classification based on active and inactive forms. A study of 753 cases.. Arch Intern Med.

[62] Garcia HH, Del Brutto OH (2003). Imaging findings in neurocysticercosis.. Acta Trop.

[63] Osborn AG, Osborn AG (1994). Infections of the brain and its linings.. Diagnostic Neuroradiology.

[64] Carpio A, Placencia M, Santillan F (1994). Proposal for a new classification of neurocysticercosis.. Can J Neurol Sci.

[65] Del Brutto OH (2005). Neurocysticercosis.. Semin Neurol.

[66] Senanayake N, Roman GC (1993). Epidemiology of epilepsy in developing countries.. Bull World Health Organ.

[67] Del Brutto OH, Wadia NH, Dumas M, Cruz M, Tsang VC, Schantz PM (1996). Proposal of diagnostic criteria for human cysticercosis and neurocysticercosis.. J Neurol Sci 1996.

