# Supplementary material for: A Systematic Review of the Frequency of Neurocyticercosis with a Focus on People with Epilepsy
Source: PLoS Negl Trop Dis. 2010 Nov 2;4(11):e870. doi: 10.1371/journal.pntd.0000870 (PMC2970544; doi:10.1371/journal.pntd.0000870)
Supplement: Flowchart S1 — PRISMA flow diagram. (0.06 MB DOC) [file pntd.0000870.s002.doc]

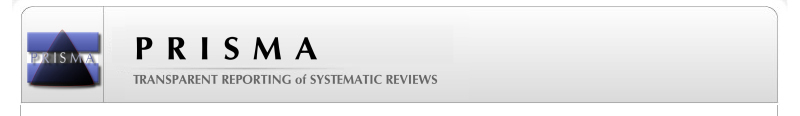
**PRISMA 2009 Flow Diagram**

**Screening**

**Included**

**Eligibility**

**Identification**

Records identified through database searching
(n = 565)

Additional records identified through other sources
(n = 9 )

Records after duplicates removed
(n = 574 )

Records screened
(n = 574 )

Records excluded
(n = 284 )

Full-text articles assessed for eligibility
(n = 290 )

Full-text articles excluded, with reasons
(n = 264 )

**Paper not found…5**

**Wrong agent….......2**

**Animal study….…..1**

**Duplicate………….3**

**Case reports……..19**

**No NCC definition.12**

**Study period……..18**

**Selection bias…….26**

**No imaging…........72**

**No original data…122**

**Not reviewed ………2**

Studies included in qualitative synthesis
(n =26 )

Studies included in quantitative synthesis (meta-analysis)
(n = 12)
